# Topology and Robustness in the *Drosophila* Segment Polarity Network

**DOI:** 10.1371/journal.pbio.0020123

**Published:** 2004-06-15

**Authors:** Nicholas T Ingolia

**Affiliations:** **1**Department of Molecular and Cellular Biology, Harvard UniversityCambridge, MassachusettsUnited States of America

## Abstract

A complex hierarchy of genetic interactions converts a single-celled Drosophila melanogaster egg into a multicellular embryo with 14 segments. Previously, von Dassow et al. reported that a mathematical model of the genetic interactions that defined the polarity of segments (the segment polarity network) was robust (von Dassow et al. 2000). As quantitative information about the system was unavailable, parameters were sampled randomly. A surprisingly large fraction of these parameter sets allowed the model to maintain and elaborate on the segment polarity pattern. This robustness is due to the positive feedback of gene products on their own expression, which induces individual cells in a model segment to adopt different stable expression states (bistability) corresponding to different cell types in the segment polarity pattern. A positive feedback loop will only yield multiple stable states when the parameters that describe it satisfy a particular inequality. By testing which random parameter sets satisfy these inequalities, I show that bistability is necessary to form the segment polarity pattern and serves as a strong predictor of which parameter sets will succeed in forming the pattern. Although the original model was robust to parameter variation, it could not reproduce the observed effects of cell division on the pattern of gene expression. I present a modified version that incorporates recent experimental evidence and does successfully mimic the consequences of cell division. The behavior of this modified model can also be understood in terms of bistability in positive feedback of gene expression. I discuss how this topological property of networks provides robust pattern formation and how large changes in parameters can change the specific pattern produced by a network.

## Abstract 2

The striped segmentation pattern of the Drosophila embryo is remarkably insensitive to variation (robust). Ingolia uses computational methods to show that this robustness results from specific positive feedback loops

## Introduction

**Figure 1:**
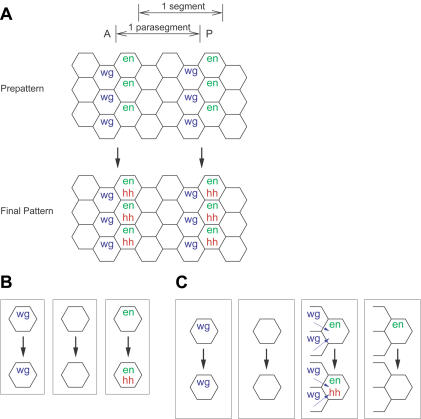
The Segment Polarity Pattern and the Behavior of Different Cells (A) Parasegments in the segment polarity pattern. The prepattern, with stripes of *wg* and *en* expression, and the final segment polarity pattern are shown. The parasegment is the basic developmental unit in the segment polarity pattern, but segment boundaries within the adult insect are offset from the parasegment boundary. (B) A simple set of rules sufficient to achieve segment polarity patterning. Cells expressing *wg* must continue to express *wg, en*-expressing cells must continue to express *en* and begin expressing *hh*, and cells expressing neither *wg* nor *en* cannot begin expressing either. (C) The behavior of isolated cells for parameter sets that form the segment polarity pattern. These are like the simple rules in (B), but *en* expression depends on a *wg*-expressing neighbor.

The network responsible for segment polarity in the Drosophila melanogaster embryo has been extensively studied. The segment polarity pattern emerges from a sequence of developmental events that each refine the pattern produced by the previous event. During the early cell cycles of the embryo, cell division is suppressed and maternal morphogens induce a transcriptional cascade of genes (the gap and pair-rule genes). These in turn create a prepattern of local expression of the segment polarity genes, genes that encode a collection of signaling molecules and transcription factors whose expression specifies the location and polarity of parasegment boundaries in the embryo. After cellularization, interactions amongst the segment polarity genes maintain narrow boundaries between parasegments as the embryo grows through cell division (Figure 1A shows how the structure of the parasegment is related to that of the morphologically defined segment). Diffusible signals from the boundaries also influence cell fates across the parasegment.

Many of the qualitative interactions between the components of the segment polarity network are known, but there is little quantitative information about the abundance of the components or the parameters that govern the reactions amongst them (DiNardo et al. 1994; Gilbert 1997; Hatini and DiNardo 2001; Sanson 2001). The existing, qualitative knowledge has been used to develop a variety of mathematical models. Some have employed Boolean idealizations (Albert and Othmer 2003), while others, including von Dassow et al., have used systems of ordinary differential equations to simulate concentrations of proteins and mRNAs (von Dassow et al. 2000; von Dassow and Odell 2002). The model requires 50 quantitative parameters such as rate constants and affinities. The equations and parameters, together with the initial conditions, specify how the protein and mRNA concentrations change over time. Von Dassow et al. tested pattern formation by picking thousands of randomly chosen parameter sets and following the evolution of the pattern from a fixed set of initial conditions. Given the large number of variables, they found that a remarkable fraction (0.5%) of parameter sets converted the prepattern into the correct, stable segment polarity pattern and concluded that the network was surprisingly robust.

I asked what general features of the model yield this robustness. As defined by von Dassow et al., the task of forming the segment polarity pattern is simple. Embryos in the model begin with a prepattern composed of a repeating unit of three stripes that encompasses four rows of cells. The first stripe expresses *wingless (wg),* the second stripe expresses *engrained (en),* and the third stripe, which is two cells wide, expresses neither. The prepattern is produced by the transient expression of gap and pair-rule genes, but maintaining and elaborating this pattern depends on the activity of *wg* and *en* and of genes that interact with them (Hatini and DiNardo 2001; Sanson 2001). For example, the *en*-expressing stripe must start to express *hedgehog (hh),* as shown in Figure 1A There is no initial *hh* expression, but the target pattern as defined by von Dassow et al. requires it to be expressed in the *en* stripe. Because EN protein induces *hh* expression, simply maintaining the initial pattern of *wg* and *en* expression suffices to produce the desired final pattern (Figure 1B) (Tabata et al. 1992).

**Figure 2:**
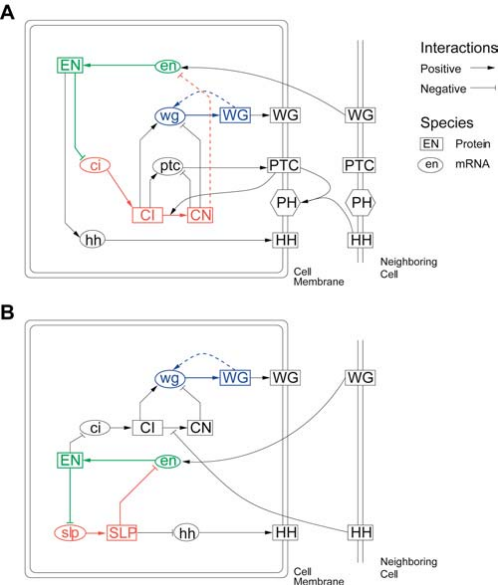
The Regulatory Networks in the Segment Polarity Models (A) The regulatory network used in the von Dassow et al. (2000) model. Dashed lines indicate interactions added by the original authors in order to achieve proper patterning, while solid lines indicate interactions based on experimental observations. The positive feedback system including *wg* is in blue, while the one involving *en* is green and red. The *en* feedback involves mutual inhibition of *en* and *ci,* so one side of the mutual inhibition scheme is drawn in green while the other is drawn in red. When the green species are active, they will repress the red ones, and vice versa. Adapted from von Dassow et al. (2000). (B) The regulatory network of the model developed here. The positive feedback systems are colored as in (A). The *en* feedback involves mutual inhibition of *slp,* however, and *ci* does not play a role in the *en* feedback system.

Thus, stable maintenance of *wg* and *en* expression levels within each individual cell will produce the segment polarity pattern. Systems in which genes induce their own expression can display multiple stable expression states, a phenomenon known as bistability, though they only do so under certain conditions (Novick and Weiner 1957; Glass and Kauffman 1973; Keller 1994; Hasty et al. 2000; Thomas and Kaufman 2001). To produce mathematical models that succeeded in converting the prepattern into the final pattern, von Dassow et al. added two interactions to their initial model of the segment polarity network. As they later noted, these created two positive feedback loops, one including *en* and the other including *wg* (Figure 2A) (von Dassow and Odell 2002). I asked whether parameter sets that can generate the segment polarity pattern are the ones that produce bistability.

**Figure 3:**
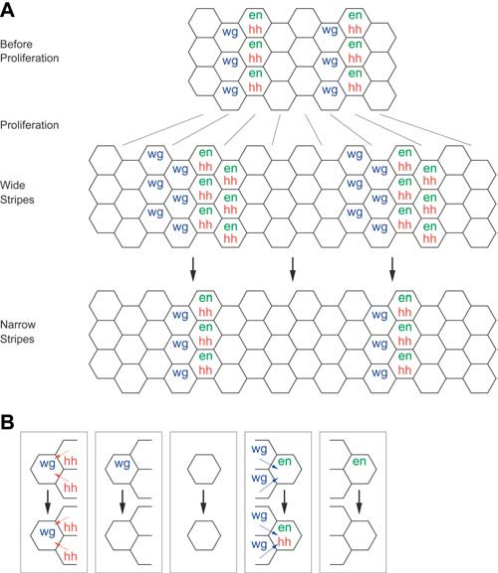
The Segment Polarity Pattern After Cell Proliferation (A) Parasegments in the segment polarity pattern during cell proliferation. During cell proliferation, each cell duplicates into two cells that initially have identical gene expression. This yields wide stripes of *wg* and *en* expression at parasegment boundaries immediately after cell proliferation. Subsequently, differences in intercellular signaling cause the stripes of *wg* and *en* narrow. (B) A simple set of rules sufficient to maintain narrow boundaries after cell proliferation. These are like the simple rules in Figure 1C, but *wg* expression also depends on a *hh*-expressing neighbor.

To address this question, I asked two questions: could modeling the behavior of individual cells reproduce the overall behavior observed by von Dassow et al., and could I produce simple rules that predicted how the individual cells would behave. When I simulated the behavior of individual cells using the von Dassow et al. model, I found that individual cells in their model can adopt three different stable states of *wg* and *en* expression. The overall pattern, and its robustness, can be simply explained as a consequence of single cells maintaining one of these expression stable states, which correspond to the three stripes of gene expression. I also devised tests that determine whether a given parameter set allows positive feedback to stably produce the desired pattern of gene expression in these cells. These allowed us to show that parameter sets that do not produce bistability almost never yield the correct pattern, whereas those that do are much more likely to produce the right segment polarity pattern. I also investigated the role of the prepattern and found that more biologically reasonable initial conditions can dramatically reduce the fraction of parameter sets that obey the bistability rules but fail to form the segment polarity pattern. Finally, I noted that the interactions of these loops do not maintain the observed segment polarity pattern after cell proliferation (Figure 3A). I modified the von Dassow scheme to incorporate recent experimental evidence and produced a model that both forms the segment polarity pattern and maintains it during cell proliferation with many random parameter sets.

## Results

I began by asking if the von Dassow et al. model could be decomposed into the properties of individual cells. The simplest hypothesis is that parameters that allow individual cells to maintain their initial state of *wg* and *en* expression will maintain the overall pattern. At the level of the cell, the parameters must allow all three types of cells to evolve from the initial conditions to the final state, and the final state must be stable. The isolated cell rules are: (1) cells that initially express *wg* must continue to do so, (2) cells that initially express *en* must continue to do so, and (3) cells that express neither *wg* nor *en* must not turn on either gene.

I began by studying the properties of *wg*-expressing cells, as WG protein is modeled as controlling *en* expression, but not vice versa (data not shown). I used the equations of von Dassow et al. to model the dynamic behavior of an individual cell, starting from the standard prepattern (von Dassow et al. 2000). I tested the isolated cell rules by simulating an individual cell in the context of signals that it would receive from its neighbors in the actual segment polarity pattern, computed assuming constant expression levels of segment polarity genes in those cells. Each parameter set that produces the overall pattern gives two behaviors that depend on the initial state of the cell; cells that are initially *wg*-expressing remain so, whereas cells that lack *wg* expression never acquire it. Thus, the *wg*-expressing stripe could retain *wg* expression while other cells in the field would not begin expressing *wg*. The precise expression levels in these two states were generally unaffected by the signals from their neighbors; in particular, HH signaling generally had no effect on *wg* expression in nearby cells (data not shown).

In the segment polarity pattern, cells on the posterior side of the *wg* stripe maintain *en* expression while cells on the anterior side of the stripe do not begin expressing *en* despite experiencing the same level of WG signaling as their neighbors on the other side of the stripe (see Figure 1A). This asymmetry requires bistability in *en* expression, at least in the context of a neighboring stripe of *wg* expression. I found that such bistability existed in working parameter sets, as long as extracellular WG exceeded a threshold concentration. Above this threshold, cells expressing *en* continue to do so, but cells that lack *en* expression do not start to express *en*. This threshold was always less than the amount of extracellular WG signal received from a neighboring stripe of high *wg* expression, which presents two *wg*-expressing cells. In a very small fraction of parameter sets, additional WG signal above the threshold could switch cells from not expressing to expressing *en*. However, when this switch was present in working models, it required WG signal from at least three *wg*-expressing neighbors. Such a switch is not seen in life, however, nor is it seen in most working parameter sets. Behaviors of isolated cells are summarized in Figure 1C.

To determine how well the isolated cell rules captured the requirements for patterning, I generated random parameter sets and tested them against the single-cell behavior rules, as well as determining whether they formed the segment polarity pattern, to see how well these correlated. Around half of randomly generated parameter sets that conform to the rules actually achieve the desired segment polarity pattern (Table 1), and parameter sets that do not satisfy these rules cannot generate the desired final pattern (with a single exception in 10,000 trials). Since the rules require cells to reach the states they exhibit in the final segment polarity pattern, it is not surprising that they are necessary. However, the strong agreement between predictions based on individual cell behavior and the observed performance of the whole system argues that the model functions because individual cells adopt one of three stable expression states to form the segment polarity pattern rather than because of the complex, collective behaviors of groups of cells.

**Table 1:**
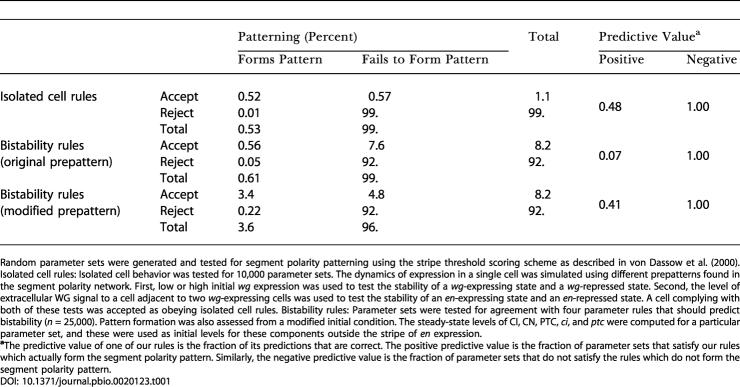
Pattern Formation and Predictive Rules in the von Dassow et al. Model

Random parameter sets were generated and tested for segment polarity patterning using the stripe threshold scoring scheme as described in von Dassow et al. (2000). Isolated cell rules: Isolated cell behavior was tested for 10,000 parameter sets. The dynamics of expression in a single cell was simulated using different prepatterns found in the segment polarity network. First, low or high initial *wg* expression was used to test the stability of a *wg*-expressing state and a *wg*-repressed state. Second, the level of extracellular WG signal to a cell adjacent to two *wg*-expressing cells was used to test the stability of an *en*-expressing state and an *en*-repressed state. A cell complying with both of these tests was accepted as obeying isolated cell rules. Bistability rules: Parameter sets were tested for agreement with four parameter rules that should predict bistability (*n* = 25,000). Pattern formation was also assessed from a modified initial condition. The steady-state levels of CI, CN, PTC, *ci*, and *ptc* were computed for a particular parameter set, and these were used as initial levels for these components outside the stripe of *en* expression

**^a^**The predictive value of one of our rules is the fraction of its predictions that are correct. The positive predictive value is the fraction of parameter sets that satisfy our rules which actually form the segment polarity pattern. Similarly, the negative predictive value is the fraction of parameter sets that do not satisfy the rules which do not form the segment polarity pattern

**Figure 4:**
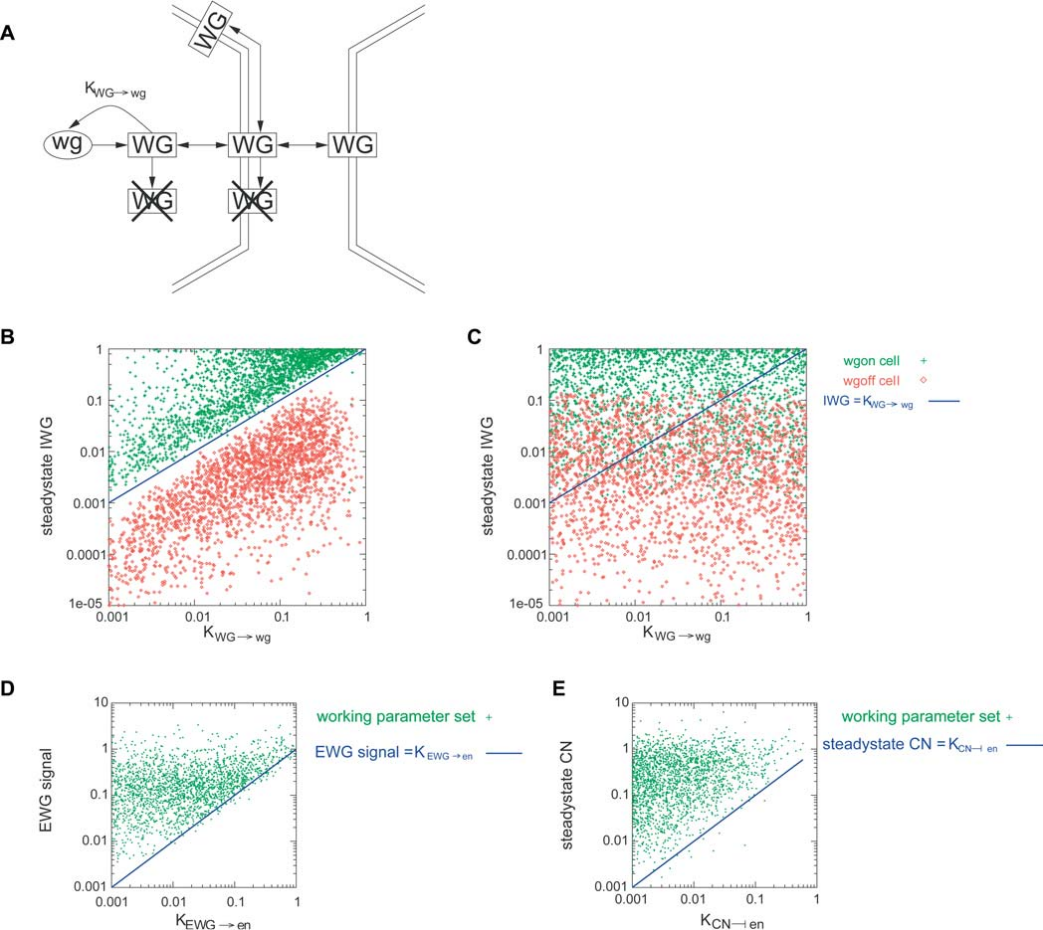
Inequalities Necessary for Bistability Are Satisfied by Working Parameter Sets (A) Subnetwork responsible for *wg* expression bistability. Levels of intercellular WG in a cell with full *wg* expression and in an adjacent cell can be computed from the transfer rates *Endo_WG_, Exo_WG_, LMxfer_WG_,* and *Mxfer_WG_*; and the decay rates *H_EWG_* and *H_IWG_*, using the linearity of WG transport processes. (B and C) Intercellular WG levels in a cell expressing *wg* (green) and in an adjacent cell (red) were plotted against K_WG→wg_, the threshold level of intercellular WG protein needed for *wg* autoactivation. In (B), parameter sets that maintain the segment polarity pattern were used, while in (C) random parameter sets were used. (D) Levels of extracellular WG signalling to a cell adjacent to two with full *wg* expression were computed as described above. These were plotted against K_EWG→*en*_, the threshold level of extracellular WG signal needed to activate *en* expression. (E) Steady-state levels of CN in the absence of *en* expression plotted against K_CN┤*en*_, the threshold level needed to repress *en* expression.

Asking whether mathematical expressions can predict the behavior of single cells and the parasegment as a whole is a more stringent test of the idea that the bistability of positive feedback loops explains these stable expression states. Whether a positive feedback loop shows bistability depends on the quantitative values of its parameters. Thus, if I can predict which sets of parameters produce bistable expression of *wg* and *en,* I can ask whether bistability in the two feedback loops is both necessary and sufficient to maintain the segment polarity pattern. The parameter sets must meet certain conditions: positive feedback must be sufficient to maintain the high-expression state, while basal or external activation must not overwhelm the low-expression state (see Protocol S1 for details). These conditions can be expressed analytically, and I devised tests to determine whether a parameter set would yield the desired bistability in both the *en* and the *wg* positive feedback loops. For instance, the amount of WG present in a cell in the high-*wg*-expression steady state was compared to K_WG→wg_, a parameter indicating the amount of intracellular WG needed for half-maximal activation of *wg* expression. I selected subnetworks within a single cell that could be largely isolated from other parts of the model (for example, see Figure 4A). I solved for approximate steady-state concentrations of components in subnetworks and compared these levels of signaling molecules to those needed to induce or repress target genes. Our derived constraints were the following.

(1) For high *wg* expression, the net level of intracellular WG must be above K_WG→wg_, the amount needed for half-maximal activation of *wg.* To maintain the pattern, expression of *wg* must be bistable, such that cells beginning with high levels of *wg* expression maintain this “on” state while those with low levels of *wg* expression remain “off.” Expression of *wg* is regulated principally by intracellular WG protein. To achieve bistability, the level of WG protein in a cell with high *wg* expression must be sufficient to activate *wg* expression. After being produced, WG protein is lost from the intracellular compartment by transport and decay processes (Figure 4A). The production and loss rates balance at a steady state, whose intercellular WG concentration I compared to K_WG→wg_ (Figure 4B).

(2) Transport of WG from a neighbor with high levels of *wg* expression cannot raise the concentration of intracellular WG above K_WG→wg_. Similarly, levels of WG protein that accumulate in cells with low *wg* expression by transport processes and basal transcription must not be high enough to activate *wg* expression in these cells. In particular, the steady-state concentration in a cell producing *wg* must exceed K_WG→wg_, but the concentrations in its non-*wg*-expressing neighbors must be below this value. Parameter sets achieving the segment polarity pattern satisfy these inequalities, as shown in Figure 4B, while randomly-generated parameter sets do not (Figure 4C).

(3) Extracellular WG from two neighbors with high levels of *wg* expression must be greater than K_*EWG*→*en*_, the amount of extracellular WG signal needed for half-maximal induction of *en* expression. Extracellular WG signaling must be sufficient to activate *en* in the absence of Cubitus Interruptus (CI) repression. The parameter K_*EWG*→*en*_ indicates the amount of extracellular WG signal needed for half-maximal *en* activation. The WG signal produced by cells in the high-*wg*-expression steady state must be strong enough to activate *en* and thus greater than K_*EWG*→*en*_. Working parameter sets satisfied this constraint (Figure 4D), while random parameter sets typically did not (data not shown).

(4) The steady-state level of the CI amino-terminal fragment (CN) must be greater than K_CN┤*en*_, the amount of CN needed for half-maximal repression of *en* expression. Repressive CI must also be sufficient to block *en* expression in cells that are near the WG stripe, but which lack *en* expression. In the absence of *en* expression, levels of CN are governed by transcriptional regulation of Patched (PTC). The equations in the model give a single steady-state level of CN. This must be greater than K_CN┤*en*_, the amount of CN needed for half-maximal repression of *en*. As shown in Figure 4E, this inequality holds for all parameter sets that form the segment polarity pattern. The interpretation of this constraint is more complicated because interactions of CI and PTC can cause persistent limit-cycle oscillations of CN about its steady-state level according to both simulations and analysis. However, this does not seem to affect our results, probably because the average level of CN across the oscillations is typically close to the steady-state level. Mutual inhibition provides two stable states, one in which *en* is expressed and represses *ci*, and one in which *ci* is expressed and maintains *en* repression.

Through such comparisons, I found that of the 0.61% of parameter sets that produced the segment polarity pattern, more than 90% were predicted to produce bistable behavior in both the *wg* and *en* positive feedback loops (see Table 1). From another perspective, the fraction of parameter sets that maintain segment polarity is enriched more than 10-fold amongst those obeying the bistability rules: 0.61% of all parameter sets form the desired pattern, but 6.8% of the parameter sets that obey the bistability rules do so. Most likely, the small fraction (0.05%) of parameter sets that form the pattern but do not obey our bistability criteria fail to do so because of approximations used in these tests. In all 12 cases, they violate only a single rule, whereas the median parameter set that does not form the segment polarity pattern violates three of the four constraints.

While 8.2% of all random parameter sets are consistent with the above restrictions, only 0.56% actually form the segment polarity pattern (see Table 1) whereas 7.6% do not. These parameter sets should maintain the segment polarity pattern, but cannot form it from the prepattern. Though the prepattern does have *en* and *wg* stripes, it lacks any expression of three regulators *(hh*, *ptc,* or *ci)* that are expressed in the final segment polarity pattern. Because the initial conditions are substantially different from the stable segment polarity pattern, there are initially large, rapid changes in the concentrations of the components that can drive the collection of cells towards a different final pattern. These early dynamics are complicated, and I could not determine simple rules that predicted which of the parameter sets that satisfied our bistability criteria would generate the segment polarity pattern starting from the initial conditions used by von Dassow et al.

The predictive value of the bistability rules is in marked contrast to the performance of the isolated cell rules, for which half the parameter sets that satisfied the rules produced the correct segment polarity pattern. I believe that the methods differ because the isolated cell rules address the dynamics by using simulations in which early expression dynamics actually occurred, at least in individual cells, rather than the steady-state comparisons of parameters used in the bistability rules. To test this possibility, I asked if I could improve the predictive value of the bistability rules by choosing different initial conditions. I focused on initial levels of CI, PTC, and CN outside the stripe of *en* expression. Increasing the initial concentrations of CI and PTC is biologically reasonable, as *ci* and *ptc* are both expressed before *en* induction (Motzny and Holmgren 1995). These two regulators constituted one of the isolated subnetworks used above. I solved for the steady-state expression levels of *ci* and *ptc* in each parameter set and used this in the initial condition for dynamic simulations with this parameter set. This change brought the prepattern in the model into better agreement with experimental results. The new initial conditions yielded a 6-fold increase in the number of parameter sets achieving the segment polarity pattern. This meant that 41% of the parameter sets meeting the bistability parameter rules actually formed the pattern from the modified prepattern (see Table 1), supporting the idea that many parameter sets obeying the bistability rules are able to form the segment polarity pattern but fail to do so from the initial pattern used by von Dassow et al. This suggests that the expression pattern of *ci* and *ptc* established by the pair-rule genes is biologically significant and plays a role in the robust formation of the final segment polarity pattern. This early expression of *ci* and *ptc* generates a prepattern that is more similar to the desired stable state.

Maintaining the narrow parasegment boundary after cell division is an important role of the segment polarity network. Even at the level of the isolated cell rules, there is a discrepancy between the behavior of the model and experimental results. Experimentally, the maintenance of *wg* expression depends on HH signal from a neighboring stripe of *en* expression, but the *wg* “on” state is unconditionally stable in the von Dassow et al. model (compare Figure 1C and Figure 3B) (Hatini and DiNardo 2001). This difficulty manifested itself when I incorporated cell division into the model. The stripe of *wg* expression should remain one cell wide as the segment widens by cell division. The daughters of cells in the *wg* stripe further from the *en* stripe will not be exposed to HH signaling and will therefore lose *wg* expression, leaving only one cell in the *wg* “on” state after each division. In the von Dassow et al. model, the independence of *wg* expression from HH, and thus *en* expression, allows both daughters of a cell in the stripe of *wg* expression to retain the *wg* “on” state. Thus, the stripe grows wider over repeated rounds of cell division rather than maintaining a narrow border at the segment boundary. Indeed, I found no parameter sets which maintained the physiological segment polarity pattern after cell division.

I wanted to modify the model so that it succeeded at this patterning task as well. Principally, I needed to make *wg* expression dependent on HH signaling (see Figure 2B). All effects of HH signaling are believed to be mediated by CI in its activating or repressive forms (Methot and Basler 2001). These regulate *wg* in the von Dassow et al. model, but CI plays another role in the *en* positive feedback loop. Constraints imposed by this second role may limit its effectiveness in regulating *wg* in response to HH signaling. Recent evidence suggests that, while EN does repress *ci* expression, *sloppy-paired (slp)* is the second factor involved in a mutual inhibition loop with *en*. I therefore removed the repression of *en* by CN and introduced mutual inhibition of *slp* and *en*, with *slp* mediating the positive effect of EN on *hh* expression (Alexandre and Vincent 2003). As all other signal transduction systems had been removed in the original model, I also removed *ptc* and allowed HH to directly inhibit the conversion of CI into CN. The interactions in this model are shown in Figure 2B. The specific equations were similar to those used by von Dassow et al., but some details were modified; for example, the exact form of the effect of CI and CN on *wg* expression was changed to account for the fact that they compete for binding to the same DNA sites (Muller and Basler 2000). I also simplified the transport processes for the intercellular signaling molecules WG and HH, which I showed play only a minor role in the original model.

**Figure 5:**
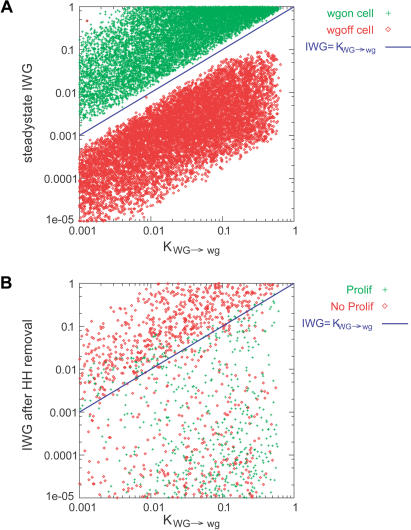
Inequalities Necessary for Bistability in the Modified Segment Polarity Model (A) Intercellular WG levels in a cell that expresses *wg* (green) and that does not express *wg* (red) were plotted against K_WG→wg_for each parameter set that forms the segment polarity pattern, as in Figure 4B. In both cases, cells are receiving maximal HH signal from two neighbors. (B) Intercellular WG levels in a cell that is expressing *wg* but is no longer receiving HH signal from any neighbors were plotted against K_WG→wg_. Parameter sets that can produce the proper pattern after proliferation, including narrow stripes of *wg* expression, are shown in green while those that fail to do so are shown in red.

This modified model can robustly form the segment polarity pattern. Taking the same approach of testing random parameter sets, I found that 9.6% could generate the segment polarity pattern. This is an 8-fold higher fraction of successful parameter sets than that seen for the von Dassow et al. model or any subsequent variants (von Dassow and Odell 2002). In order to test whether this was a result of bistability in *wg* and *en* expression, I developed bistability rules for the modified model. These rules require the following: (1) the amount of intercellular WG in a cell with high *wg* expression must be enough to activate *wg* expression; (2) the amount of intracellular WG in a cell with low *wg* expression, but receiving strong HH signaling, must not be high enough to activate *wg* expression; (3) the amount of EN in a cell with low *slp* expression and high WG signaling from neighbors must be enough to repress *slp* expression; and (4) the amount of EN in a cell with high *slp* expression, but high WG signaling from neighbors, must not be sufficient to repress *slp.* Nearly all working parameter sets obeyed these rules, as I found for the von Dassow et al. model (see Figure 5A). They were even better predictors of working parameter sets than the parameter rules in the original model; nearly half of random parameter sets that are consistent with these rules form the proper pattern (Table 2).

**Table 2:**
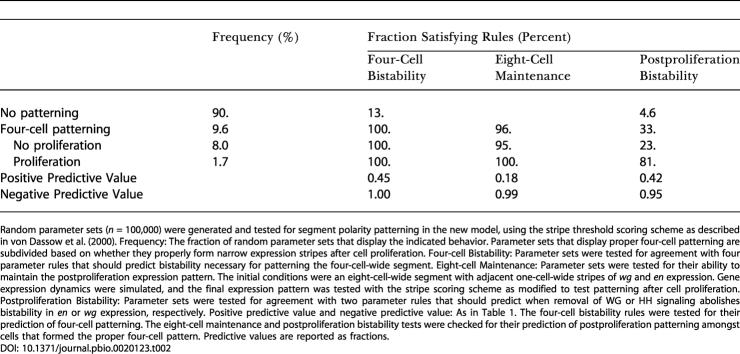
Pattern Formation and Predictive Rules in the Modified Model

Random parameter sets (*n* = 100,000) were generated and tested for segment polarity patterning in the new model, using the stripe threshold scoring scheme as described in von Dassow et al. (2000). Frequency: The fraction of random parameter sets that display the indicated behavior. Parameter sets that display proper four-cell patterning are subdivided based on whether they properly form narrow expression stripes after cell proliferation. Four-cell Bistability: Parameter sets were tested for agreement with four parameter rules that should predict bistability necessary for patterning the four-cell-wide segment. Eight-cell Maintenance: Parameter sets were tested for their ability to maintain the postproliferation expression pattern. The initial conditions were an eight-cell-wide segment with adjacent one-cell-wide stripes of *wg* and *en* expression. Gene expression dynamics were simulated, and the final expression pattern was tested with the stripe scoring scheme as modified to test patterning after cell proliferation. Postproliferation Bistability: Parameter sets were tested for agreement with two parameter rules that should predict when removal of WG or HH signaling abolishes bistability in *en* or *wg* expression, respectively. Positive predictive value and negative predictive value: As in Table 1. The four-cell bistability rules were tested for their prediction of four-cell patterning. The eight-cell maintenance and postproliferation bistability tests were checked for their prediction of postproliferation patterning amongst cells that formed the proper four-cell pattern. Predictive values are reported as fractions

Rules 1 and 2 are exactly analogous to bistability rules for the original model that ensure bistability in *wg* expression. The only change is the inclusion of HH signaling, which regulates *wg* expression in the modified model. Because *ci* is responsible for transducing the HH signal, I needed to find a different mutual inhibition partner for *en*; recent experiments implicated *slp* in this process. Rules 3 and 4 are similar to the bistability rules that ensure mutual repression of *en* and *ci* expression in the original model. They require high *en* expression to be strong enough to repress *slp* and vice versa. This ensures that either of the two states of the mutual inhibition switch is stable, and so *en* expression is bistable.

In addition to maintaining the initial segment polarity pattern, the modified model is also capable of producing the proper pattern after cell division. Fully 1.7% of random parameter sets yielded the desired narrow stripes of gene expression after division, showing that this feature of the modified model is also robust (see Table 2). I investigated whether bistability of *wg* and *en* expression also explained which parameter sets could produce the proper pattern after cell proliferation. Achieving this pattern requires two steps: as cells proliferate, half of the daughters of *wg*- or *en*-expressing cells must turn off these genes, and the resulting pattern must be stable over time. The criteria for the stability of the pattern after proliferation are quite similar to those for the original pattern. In fact, nearly all parameter sets that form the original pattern can also maintain the eight-cell-wide segment pattern with one-cell-wide stripes if this pattern is used as an initial condition (see Table 2). Thus, parameter sets that fail to generate this pattern after cell proliferation must have difficulty reaching the proper pattern rather than maintaining it once produced. The more important constraint, as discussed above, is that *wg* and *en* expression must fade as cells move away from the mutual reinforcement at the boundary. I devised two additional rules based on this mutual dependence: (5) the amount of intracellular WG in a cell with high *wg* expression, but receiving no HH signaling, must not be sufficient to maintain *wg* expression (Figure 5B); and (6) the amount of SLP in a cell with initially high *en* expression that stops receiving strong WG signaling must be enough to repress *en* expression. Only one row of cells on either side of the parasegment border will be receiving WG or HH signals across the boundary. Rules 5 and 6 ensure that this signaling is necessary to maintain *en* and *wg* expression, so daughter cells born away from the boundary lose *en* or *wg* expression. This dependence is the mechanism by which the modified model maintains narrow stripes of segment polarity gene expression after cell division.

These rules are reasonable predictors for proper behavior during cell proliferation. However, there are a substantial fraction of parameter sets that work despite breaking one or both rules, as well as many which obey them yet cannot produce the proper pattern after one round of cell division. Some of these difficulties probably result from the dynamic nature of the underlying process. As discussed above, bistability rules such as these can determine when a particular expression state is stable, but it is much harder to determine which stable state will be reached for a given initial condition. Thus, it is possible to predict when a parameter set will be able to maintain the final postproliferation pattern, but it is much harder to determine when it will reach this pattern from the expression state immediately following proliferation. This does not explain why there are parameter sets that do not obey rules 5 and 6 but nonetheless give narrow stripes of gene expression after cell division. Those parameter sets expose limits in the approximations used to develop the cell bistability rules. There may be small but important interactions between different feedback loops within a single cell, or perhaps some aspect of intercellular signaling is more complicated than the simple binary model employed in the bistability rules.

## Discussion

I have shown that individual cells in the segment polarity model can adopt three distinct expression states, influenced by signals from their neighbors. I have also presented evidence that positive feedback in the model produces these states. The importance of autoregulation in establishing distinct expression states has been recognized in this system before (Heemskerk et al. 1991). In general, positive feedback can produce discrete stable expression states which are insensitive to small changes in parameters or initial conditions (Thomas and Kaufman 2001). This explains the robustness of the segment polarity patterns in the models. The ways in which intercellular signals impinge on autoregulatory loops will determine which expression patterns are possible. In a field of cells with bistable expression states, the overall pattern is just a specification of a particular expression state for each cell in the field. When signals produced by cells in the pattern are consistent with the states of neighbors receiving them, then this pattern will be a stable steady state. In the segment polarity pattern, there is a stripe of high *en* expression posterior to the stripe of *wg* expression, but one with low *en* expression anterior to it. The stripe of *wg* expression produces a signal that is strong enough to maintain the high *en* expression state, but does not induce *en* expression in cells that do not initially express it. Thus, the states of the cells neighboring the stripe of *wg* expression are consistent with the signals it produces. Our modifications to the model changed the effect of HH on the *wg* autoregulatory loop. This destabilized the pattern of wide stripes of *wg* expression resulting from cell proliferation, retaining the desired pattern with narrow *wg* expression as a stable pattern. Because the wide-stripe pattern was no longer stable, the model did not become trapped in this state following cell division. Many parameter sets instead progressed to the narrow-stripe pattern.

The approach I have taken can be generally applied to models of complicated genetic or biochemical networks. I isolated small subnetworks, chosen to be maximally insulated from the rest of the system, and studied their behavior in isolation. This let us understand the principles that allow the entire network to function. I verified this understanding by creating tests for the behavior of the subnetworks and showing that these were powerful predictive tools for the performance of the entire network. This sort of decomposition is also useful in combination with quantitative phenomenological descriptions of subnetwork behaviors. Recent experimental studies provide such descriptions for a number of biological systems, including vertebrate homologues of the *wg* signal transduction system (Bagowski and Ferrell 2001; Bhalla et al. 2002; Lee et al. 2003). These could replace subnetworks in a larger model, tying the model more closely to biological evidence and showing how the subnetwork affects the larger system in which it functions.

The robustness of the segment polarity network is a result of the fact that the desired pattern is a stable steady state. In a system of ordinary differential equations, such as the models described here, such states correspond to stable fixed points. These are generic features of such systems; small changes in parameters or initial conditions will not change them qualitatively. This can be seen in the bistability rules I developed. They are inequality constraints, so they carve out a volume of parameter space in which parameter sets can maintain the segment polarity pattern. In our analysis, I focused on robustness against changing parameters, which correspond to genetic alterations that change quantitative values of reaction parameters. In the real world, stochastic and environmental perturbations in the system may play at least as large a role.

One important question is the extent to which the behavior of a network is determined by its topology, as opposed to quantitative details. The network topology is just the set of interactions in the network, along with their signs. This information is accessible to standard, qualitative biological experiments. Topology limits the possible behaviors of a regulatory network. Positive feedback, which is a topological property, is necessary for multiple stable states (Thomas and Kaufman 2001). Without such autoregulatory loops, all cells would eventually return to the same state after inducing signals are removed. Thus, positive feedback is particularly important in development and differentiation, when many different cell fates are permanently specified. However, quantitative details still have a large influence on network behavior. I held network topology constant while testing random parameter sets, which corresponds to changing quantitative details. Most random parameter sets did not form the segment polarity pattern because they did not display the proper stable states, despite having a topology that was capable of forming the segment polarity pattern. Quantitative details select a particular behavior from the repertoire of behaviors that are accessible from a given network topology. This same phenomenon has been shown experimentally in synthetic genetic networks, where a single topology can give rise to different behaviors when transcription factors and their binding sites are varied (Guet et al. 2002).

These examples show how changes in the quantitative details of a regulatory network can result in qualitatively different behaviors. This could explain how pattern formation can be evolvable; mutations which cause large shifts in a critical parameter could cause a network to form a different pattern corresponding to a new stable state. The altered pattern would still correspond to a stable fixed point, so it would also be robust against various kinds of perturbations. This offers a mechanism that could produce new patterns without nonfunctional intermediates and without events such as the creation of a new protein–protein interaction.

## Materials and Methods

### 

The model employed a system of differential equations described by von Dassow et al. (2000). The correspondence between variables and parameters in our model and theirs is in Table 3. Simulations and numerical approximations were performed using the GNU Scientific Library (Galassi et al. 2002).

**Table 3:**
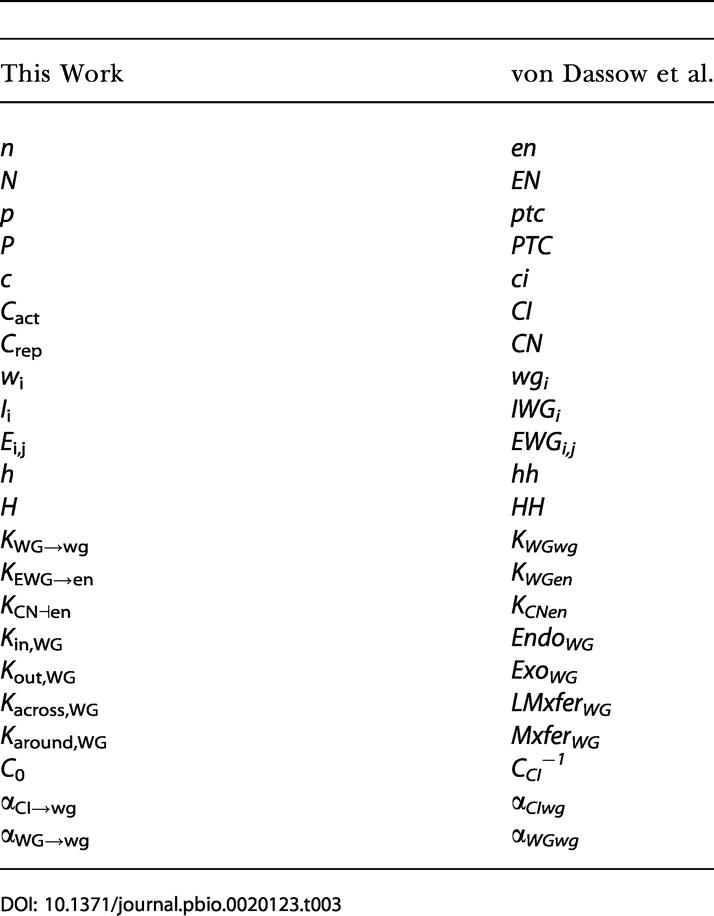
Variables in This Work and von Dassow et al. (2000)

#### Isolated cell rule simulations

Isolated cell rules were tested by simulations in which the dynamics of an individual cell were modeled using the same equations that govern each cell in the segment for the full segment polarity network.

Since WG protein diffused between cells as well as moving into and out of a given cell, it was important to account for the diffusion of WG even in isolated cell simulations. The level of *wg* mRNA in a cell is represented by *w_i_*. Once translated from *wg* mRNA, WG protein diffuses between the intracellular pool, represented by *I_i_*, and extracellular pools on each face *j* of the cell, *E*_*i*,*j*_. Extracellular WG can exchange between faces of the same cell and between opposing faces of adjacent cells.



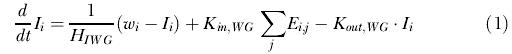





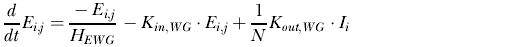





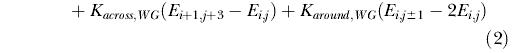



The parameters *H_IWG_* and *H_EWG_* are the half-lives of WG in the intracellular and extracellular pools, respectively. The diffusion parameters *K_out,WG_*, *K_across,WG_*, *K_around,WG_*, and *K_in,WG_* are the rate constants for the first order of exchange of WG between the intracellular and extracellular pools and between different cell faces. These are linear equations, so it is possible to solve for the steady-state levels of *I_i_* and *E_i,j_* as a function of the *w_i_s*, which control WG production, by inverting a matrix of transport and decay rates. In the segment polarity pattern, particularly, there is just one *wg*-expressing cell in the periodic pattern of four cells. So, I take *w_on_* for one cell and *w_off_* for the other three in the periodic unit. All WG protein is initially intracellular, but it moves to extracellular faces by a roughly first-order process with time constant *k* = *K_in,WG_*+*H*^−1^_*EWG*_. Therefore, I used *E_i,j_*(*t*) = (1 −*e^kt^*) · E˜_*i,j*_ as the amount of WG protein on neighboring cells for the isolated cell simulations.

To verify bistability of *wg* expression, I simulated a single cell with no HH signaling from its neighbors. I calculated the amount of WG protein expected to be present on neighbors by an iterative process. Starting with *w_on,0_* = 1 and *w_off,0_* = 0, I computed the steady-state extracellular WG protein E˜*_i,j_*(*w_on_*,*w_off_*) presented by the neighbors of the cell expressing *wg* and used these in simulating a cell with initial *w* = 1. Similarly, I computed the amount of WG protein on neighbors of a cell next to the stripe of *wg* expression and used this in simulating a cell with initial *w* = 0. The final values of *w* in those two cells were used as *w*_*on,i*+1_ and *w*_*off,i*+1_ to compute the levels of extracellular WG protein for the next iteration. This process quickly converged, and I took the resulting *w* values as *w_on_* and *w_off_*. I verified that *w_on_* was above 0.1, the expression level threshold used in scoring pattern formation, and that *w_off_* was below 0.1.

I then used the same levels of extracellular WG protein, computed from *w_on_* and *w_off_*, to simulate a cell next to a stripe of *wg* expression. I used initial *en* mRNA and protein levels of 1 or 0 and ensured that, at the end of the simulation period, the former cell had *en* expression levels over the threshold but the latter did not. Finally, I verified that a cell with high initial *wg* mRNA but low initial *en* mRNA, receiving signals as if it were in the stripe of *wg* expression, still had low *en* expression at the end of the simulation.

#### Bistability parameter rules

These equations make repeated use of a particular equation form representing saturable and cooperative action of a protein, for instance as a transcriptional activator. In general, the amount of activation, Φ, as a function of the concentration of activator, *x*, is



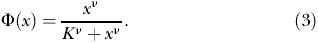



Here, *K* indicates the concentration of activator needed for half-maximal activation; it is essentially an affinity of the activator for its target. The parameter ν controls the degree of cooperativity in activator function, with large values of ν giving stronger cooperativity. The function produces sigmoidal curves which asymptotically approach 1 when *x* is large relative to *K*. In the model, there is a different Φ for each instance of transcriptional regulation controlled by an affinity parameter *K* and a cooperativity parameter ν for that interaction. For instance, the activation of *en* by extracellular WG is controlled by K_*EWG*→*en*_, which indicates the amount of extracellular WG needed for half-maximal activation, and by ν_*EWG*→*en*_, which determines how cooperative the activation is.

#### *ci* and *ptc* subnetwork

I designed parameter rules for bistability by analyzing different subnetworks in the model and solving for steady states consistent with bistability from positive feedback. I solved for the stationary state of the *ptc* and *ci* subnetwork in the absence of *en* expression. The concentrations of *ptc* and *ci* mRNAs are *p* and *c*, and the concentrations of PTC protein, activating CI protein, and repressive CN are *P*, *C_act_*, and *C_rep_*. The equations governing this system, entirely contained within a single cell, are



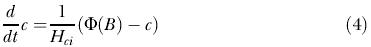





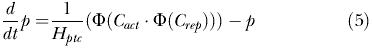





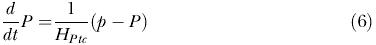





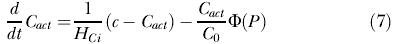





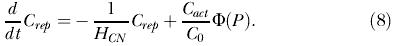



The affinity and cooperativity parameters for each Φ have been suppressed for clarity. The parameters *H_ci_* and *H_ptc_* are the half-lives of *ci* and *ptc* mRNAs, and similarly the parameters *H_Ptc_*, *H_Ci_*, and *H_CN_* are the half-lives for the protein species. The level of Bicoid, a constitutive activator of *ci* expression, is indicated by the parameter *B*. Finally, *C*_0_ is an affinity parameter for the cleavage of CI by PTC. To find the stationary state, I solve for the simultaneous zero of all five equations. Two variables, *c* and *P*, can be trivially eliminated. The remaining three equations in three variables always yielded a unique stationary state. The level of CN at this state, C˜_*rep*_, was compared to K_CN┤*en*_, the amount needed for half-maximal repression of *en* expression (parameter rule 4).

The levels of CI and CN were also used to compute their influence on *wg* expression. The strength of this activation was indicated by β, a single term encompassing activation by CI and repression by CN.



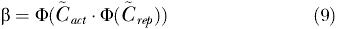



The only parameters in this expression are the affinity and cooperativity parameters for each Φ.

#### WG and its effect on *en*

Levels of *wg* mRNA in the *i*th cell, *w_i_*, are governed by β, which indicates the influence of CI and CN on *wg* expression, and by *I_i_*, the amount of intracellular WG in the cell.



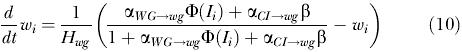



In addition to affinity and cooperativity parameters for each Φ, and *H_wg_*, the half-life of *wg* mRNA, there are scalars α_*CI*→*wg*_ and α_*WG*→*wg*_, which determine the relative strengths of CI/CN and WG influences on *wg* expression. When *I_i_*>*K*_*WG*→*wg*_, then Φ(*I_i_*) will be large and *wg* expression high. I computed steady-state intracellular and extracellular WG protein levels as a function of *wg* expression as described above for the isolated cell rules.

Bistability requires that intracellular WG levels in a *wg*-expressing cell remain high enough to maintain *wg* expression. I computed successive approximations to steady-state levels of *wg* mRNA and protein. I found *I˜*_*w*=1_=*I_i_*(*w_i_*=1) by setting 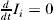 and then found *w˜*_*on*_=*w_i_*(*I_i_*=*I˜*_*w*=1_) by setting 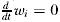. I then required that *I˜*_*on*_=*I_i_*(*w_i_*=*w˜* *_on_*)>*K*_*WG*→*wg*_, meaning that the level of intracellular WG is sufficient to maintain *wg* expression (parameter rule 1). I found no cases in which this much faster test gave different results than actually solving the self-consistent equations for 
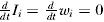
. Bistability also requires that a cell not initially expressing *wg* must not be activated by WG from a neighboring cell. I used *w˜*_*on*_ to compute the amount of intracellular WG in a cell next to the *wg* stripe but not itself expressing *wg*, *I˜* *_nbr_*=*I*_*i*+1_(*w_i_*=*w˜*_*on*_,*w*_*i*+1_=0), and found *w˜* *_off_*=*w*_*i*+1_(*I*_*i*+1_=*I˜*_*nbr*_) and *I˜*_*off*_=*I*_*i*+1_(*w*_*i*+1_=*w˜*_*off*_). I then verified that *I˜*_*nbr*_+*I˜*_*off*_<*K*_*WG*→*wg*_, meaning that the sum of intracellular WG transported into a *wg* “off” neighbor and the intracellular WG produced by the *wg* “off” neighbor is not enough to activate *wg* expression (parameter rule 2). Finally, I find levels of extracellular WG signaling *E˜*_*on*,*j*_ and *E˜*_*off*,*j*_ in the same manner as *I˜*_*on*_ and *I˜*_*off*_, respectively. These are used to ensure that the level of extracellular WG signal received by a cell in the *en* stripe is Σ*E˜*>*K*_*EWG*→*en*_ (parameter rule 3).

#### Modified initial conditions

The modified initial conditions were generated by solving for the steady state of the CI and PTC subnetwork as described above. This yielded steady-state values *c˜*, *C˜*_*rep*_, *C˜*_*act*_, *p˜*, and *P˜*, which were used for the initial conditions in the stripe of *wg* expression and in the stripe expressing neither *wg* nor *en*. Initial conditions for components of the CI and PTC subnetwork in the stripe of *en* expression were kept at 0.

The modified initial conditions also used steady-state levels of intracellular and extracellular WG protein. The steady-state *I˜*_*i*_ and *E˜*_*i*,*j*_ values were computed as described above under the assumption of a single column of cells with maximal *wg* expression and three columns with no *wg* expression. This latter change had a very modest impact on the fraction of parameter sets which formed the segment polarity pattern, and I did not pursue it further.

#### Modified model

The equations governing the modified model were similar in form to those in the original model. In addition to using the functional form Φ(*x*), I employed a related functional form Ψ(*x_r_*,*x_a_*) that represents the effects of an activator and a repressor that compete with equal affinity for a common binding site.



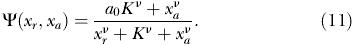



Again, *K* is essentially an affinity parameter and ν controls the cooperativity of the process. The *a_0_* term indicates the basal expression level, seen when neither activator nor repressor is acting. This functional form is used to express the effect of repressive CN and activating CI on *wg* expression. I also used it to represent the effect of intracellular WG activator with basal *wg* transcription, setting the repressor term *x_r_*=0.

In addition to the dynamic variables described above, levels of *en* mRNA and EN protein are given by *n* and *N*, and levels of *slp* mRNA and SLP protein are given by *s* and *S*, respectively. The affinity, cooperativity, and basal transcription parameters are suppressed throughout for clarity. As nearly all dynamic variables are in the same cell, subscripts that index concentrations within a given cell are also omitted. In the two equations that involve intercellular signaling, a term *E¯*_*Nbr*_ or *H¯*_*Nbr*_ indicates the sum of extracellular WG or HH on neighboring cells, respectively; this is equivalent to the average without a normalization for the number of cells.



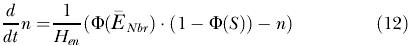





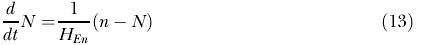





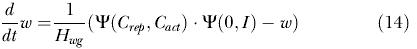





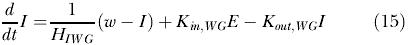





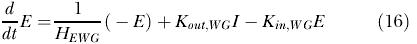





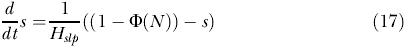





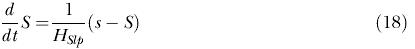





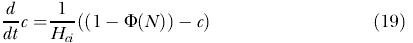





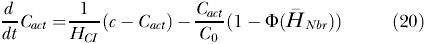





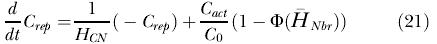





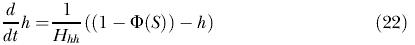





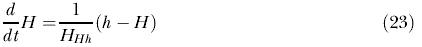



Initial conditions were *n*=*N*=1 in the stripe of *en* expression, *w*=*I*=1 in the stripe of *wg* expression, and *s*=*S*=1 in the two-cell-wide stripe expressing neither *en* nor *wg*. As in the original model, cell proliferation was accomplished by doubling the grid size and copying the dynamic variables from each cell into two adjacent cells in the new grid.

#### Bistability parameter rules

Steady-state levels *I˜* *_on_* and *I˜*_*off*_ were computed similarly to the way described for the original model. I assumed maximal *ci* expression, *c*=1, and maximal HH signal from two neighbors, *H¯*_*Nbr*_=2, in computing the steady-state levels *C˜*_*act*_ and *C˜*_*rep*_. As there was no intercellular transport of WG in the modified model, I needed to worry only about basal and activated *wg* expression in a single cell and did not need to consider intercellular transport. To check parameter rules 1 and 2, I simply compared the two steady-state levels *I˜*_*on*_ and *I˜*_*off*_ to K_*WG*→*wg*_.

I computed *E˜*_*w*=1_ for *c*=1 and *H¯*_*Nbr*=2_ to account for WG signaling in *en* expression. I then found *N˜*_*S*=0_ using *E¯*_*Nbr*_=2*E˜*_*w*=1_ to represent maximal WG signaling from two neighbors and *S*=0, no *slp* expression, in the steady-state equation *n˜*=*N˜*=Φ(*E¯*_*Nbr*_)·(1−Φ(*S*)). I used this to compute *S˜*_*off*_ using the steady-state equation *s˜*=*S˜*=(1−Φ(N)). Finally, I used *S˜*_*off*_ and *E˜*_*w*=1_ to find *N˜*_*on*_ in the *en* steady-state equation. I compared *N˜*>*K*_*EN*┤*slp*_ to ensure that steady-state levels of EN were sufficient to repress *slp* expression.

Similarly, I found *N˜*_*S*=1_ using the steady-state *en* equation and used this to find *S˜*_*on*_ using the steady-state *slp* equation. The *S˜*_*on*_ was then used to find *N˜*_*off*_, and I required that *N˜*_*off*_<*K*_*EN*┤*slp*_. This ensured that repressed levels of *en* expression were not sufficient to repress *slp* expression.

To test that *wg* expression was dependent on HH signaling, I first found *I˜*_*on*_ as described before. I also computed *C˜*_*act*_ and *C˜*_*rep*_ using *c*=1 but *H¯*_*Nbr*_=0, representing a loss of HH signaling. I then used *I˜*_*on*_ and the new *C˜*_*act*_ and *C˜*_*rep*_ to find *w˜*_*H*=0_ and *I˜*_*H*=0_ with the steady state *wg* equation. I then found *w˜*_*on*→*off*_ and *I˜*_*on*→*off*_ using the steady state *wg* equation, the new *H*=0 values for *C˜*_*act*_ and *C˜*_*rep*_, and *I˜*_*H*=0_. Finally, I verified that *I˜*_*on*→*off*_<*K*_*WG*→*wg*_, which ensure that *wg* autoactivation is not sufficient to maintain its expression after HH signaling is removed.

To check whether *en* expression was dependent on WG signaling, I started with *N˜*_*on*_ and *S˜*_*off*_ as described above. I found *E˜*_*off*_ in the same way in which I found *I˜*_*off*_ and used *E¯*_*Nbr*,*off*_=6*E˜*_*off*_. I used this new level of WG signaling to find *N˜*_*on*→*off*_ with the steady state *en* equation, and then used this value to find *S˜*_*off*→*on*_ with the steady-state *slp* equation. To verify parameter rule 6, I checked that *S˜*_*off*→*on*_>*K*_*SLP*┤*en*_, ensuring that the unrepressed level of *slp* expression can block *en* expression.

## Supporting Information

Protocol S1 Bistability in *wg* Expression Additional background and explanation of bistability in gene expression.
(109 KB PDF).

### Accession Numbers

The FlyBase (http://flybase.bio.indiana.edu/) accession numbers for the genes discussed in this paper are *ci* (FBgn0004859), *en* (FBgn0000577), *hh* (FBgn0004644), *ptc* (FBgn0003892), *slp* (FBgn0003430 and FBgn0004567), and *wg* (FBgn0004009).

## Abbreviations

Ci: Cubitus Interruptus
CN: Cubitus Interruptus amino-terminal fragment
En: Engrailed
Hh: Hedgehog
Ptc: Patched
Slp: Sloppy-Paired
Wg: wingless.
