# Supplementary material for: Topology and Robustness in the Drosophila Segment Polarity Network
Source: PLoS Biol. 2004 Jun 15;2(6):e123. doi: 10.1371/journal.pbio.0020123 (PMC423129; doi:10.1371/journal.pbio.0020123)

Bistability in *wg* Expression

Bistability arises when a gene positively regulates its own expression in a cooperative manner. This can be seen in a simplified model of *wg* regulation, in which w, the level of *wg* mRNA, is regulated by W, the amount of WG protein. The concentration of *wg* mRNA and WG protein will be normalized so that the $w = 1$ and $W = 1$ for the maximum possible *wg* expression. I will assume first-order decay for both the mRNA and protein, at rates H_{wg} and H_{WG} proportional to their half-lives. Protein will be translated from mRNA at a rate directly proportional to the amount of mRNA. Transcription will be controlled by WG protein in a highly cooperative manner, with an effective Hill coefficient of four. In the absence of any WG protein, there will be basal transcription at a rate b. The amount of WG protein needed for half-maximal activation is represented by K, which is effectively an affinity of the protein for its target site. The equations for the dynamics of mRNA and protein levels are

$$\frac{d}{dt}w = -\frac{w}{H_{wg}} + \frac{bK^4 + W^4}{K^4 + W^4} \quad \text{(S1)}$$

$$\frac{d}{dt}W = -\frac{1}{H_{WG}}(W - w) \quad \text{(S2)}$$

The state of a cell is the level of *wg* mRNA and protein. We can represent this state as (W, w), a point on a phase plane. The equations S1 and S2 indicate how the levels of mRNA and protein will change as a function of the current state. As the cell's state changes over time, it traces out a curve in the phase plane. In order to understand what these curves look like, we want to know whether levels of mRNA and protein are increasing or decreasing in some state; this is determined by the sign of $\frac{d}{dt}w$ and $\frac{d}{dt}W$. We first draw the curves along which one of the dynamic equations is zero, called the nullcline. This will separate regions in which that variable is increasing from regions in which it is decreasing. Physically, the amount of WG protein produced at a given level of *wg* expression can be read from the blue line in Figure S1A, while the amount of *wg* expression that results from a given level of WG protein is depicted by the red line. In Figure S1A, $\frac{d}{dt}W > 0$ to the left of the blue line, meaning that W increases and the cell state moves to the right; similarly, to the right of the blue line, the cell state moves left. This analysis allows us to draw arrows indicating how the state changes for each point in the plane. There are three points where the lines cross and $\frac{d}{dt}w = \frac{d}{dt}W = 0$. These are stationary states of the system—neither w nor W changes. Only the low and high ones are stable, however. All arrows around the stable stationary states point inward, meaning that they attract all nearby states. In the middle state, points below and to the left will move further away, as will those above and to the right. Almost every tiny displacement from this stable state will be amplified, so the state is not stable against

even the smallest perturbations. There are not always three crossings of these lines. As K increases, more WG protein is required to activate *wg* expression. Eventually, the amount of WG protein needed to sustain high *wg* expression is more than the amount produced at high *wg* expression. This is depicted graphically in Figure S1B, where the upper crossings of the nullclines vanish as K increases from 0.5 to 0.7. The lower stable stationary state will vanish in a similar way as the basal transcription rate, b, increases.

Figure S1. Phase Portraits of a Bistable Positive Feedback Loop

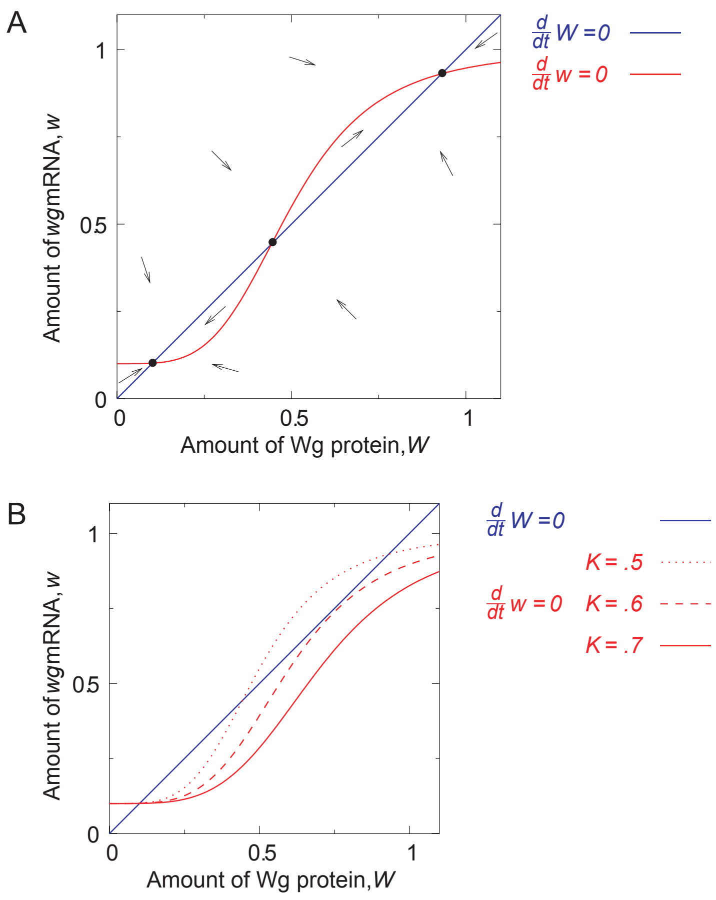

Supplement: Protocol S1 — Additional background and explanation of bistability in gene expression. (109 KB PDF). [file pbio.0020123.sd001.pdf]
